# Plasma level of miR‐99b may serve as potential diagnostic and short‐term prognostic markers in patients with acute cerebral infarction

**DOI:** 10.1002/jcla.23093

**Published:** 2020-01-22

**Authors:** Xiaomu Wu, Xiaohong Zhang, Dongqing Li, Ziyang Zhu

**Affiliations:** ^1^ Department of Neurology The Central Hospital of Wuhan Wuhan China; ^2^ Department of Neurology Wuhan No. 6 Hospital Wuhan China; ^3^ Department of Respiratory Critical Wuhan No. 6 Hospital Wuhan China

**Keywords:** acute cerebral infarction, diagnosis, miR‐99b, prognosis

## Abstract

**Objective:**

The aim of the present study is to explore the potential diagnostic and prognostic value of plasma levels of miR‐99 family for patients with acute cerebral infarction (ACI).

**Methods:**

A total of 112 patients who have been diagnosed with ACI were enrolled in this study, and 112 healthy volunteers were served as the controls. The plasma of the patients and controls were collected, and total RNAs were isolated, and the expression levels of miR‐99a, miR‐99b, and miR‐100 in the plasma of patients and controls were compared determined by RT‐qPCR methods; moreover, the receiver operating characteristic (ROC) curve has been drawn to determine whether the plasma levels of miR‐99b can distinguish patients with ACI from the controls; furthermore, the short‐term prognosis of the patients was evaluated by glasgow outcome scale (GOS), and the correlation between the plasma levels of miR‐99b and the GOS of the patients was evaluated. Finally, the correlation between the plasma level of miR‐99 and VEGF of ACI patients was analyzed.

**Results:**

It was observed that miR‐99b was significantly decreased in the plasma of ACI patients compared with the healthy controls (*P* < .01), while the plasma levels of miR‐99a and miR‐100 showed no significant differences between the patients with ACI and the healthy controls; moreover, the area under the curve (AUC) of miR‐99b for the diagnosis of ACI was 0.8882 (95% confidence interval (CI), 0.8451‐0.9313), suggesting that plasma level of miR‐99b is a sensitive marker to distinguish patients with ACI from the healthy volunteers; furthermore, the serum level of miR‐99b was negatively correlated with GOS score of the patients (*r *= −.56, *P* < .001); finally, the plasma level of miR‐99b was negatively correlated with the levels of VEGF (*r* = −.3013, *P* = .0012).

**Conclusion:**

miR‐99b was down‐regulated in plasma of patients with ACI, and plasma level of miR‐99b may be a potential diagnostic and prognostic marker for the diagnosis and treatment of ACI.

## 1 INTRODUCTION

Stroke is one of the leading causes of death worldwide.^1^, ^2^ Strokes can be classified into ischemic and hemorrhagic, and the ischemic stroke can lead to acute cerebral infarction (ACI) in the brain tissue if the local blood flow has not been recovered in a short period of time.^3^, ^4^ Results of previous studies indicated that if no rapid clinical decisions were made, ACI can lead to serious sequelae, for example, disability even death.^5^, ^6^, ^7^ Thus, early diagnosis of ACI is of great importance to improve the therapeutic efficacy and decrease the potential risk of sequelae and mortality. At present, the diagnosis of stroke was mainly depended on the traditional neurological imaging methods, for example, magnetic resonance imaging (MRI) and computed tomography (CT) capabilities^7^; however, not all hospitals were equipped with the equipment, and many patients, especially in the developing countries, cannot afford the high costs. On the other hand, the examination of the circulating biomarkers has now been considered as fast and inexpensive methods for the diagnosis of disease or evaluation of the clinical outcomes.^8^, ^9^ Therefore, to identify novel circulating diagnostic and prognostic marker for ACI is in a great demand.

MicroRNAs (miRNAs or miR) belong to the family of the non‐coding RNAs. miRNAs were often short in length (about 18‐22 nucleotides), and they can negatively regulate the expression of their target genes on post‐transcriptional levels.^10^, ^11^ The roles of miRNAs in ACI have also been reported in many previous studies, and the diagnostic and prognostic value of some circulating miRNAs, for example, miR‐124 and miR‐29b have also been discussed.^11^, ^12^

The roles of miR‐99 miRNA family (including miR‐99a, miR‐99b and miR‐100) in cerebrovascular diseases have been reported previously.^13^, ^14^, ^15^ Based on the results of a previous animal study, the level of miR‐99b in the peripheral blood was down‐regulated the brain of rat ACI models,^15^ however, whether miR‐99b could serve as a plasma diagnostic and prognostic marker remains unclear. In the present study, the diagnostic and prognostic value of plasma miR‐99 in ACI will be evaluated; and also, the relationship between plasma level of miR‐99 and angiogenic factor VEGF will be explored.

## 2 MATERIALS AND METHODS

A total number of 112 patients that diagnosed as ACI at the Department of Neurology, The Central Hospital of Wuhan, between March 2018 and September 2018 were enrolled in this study, and 112 healthy volunteers were also enrolled as the control group. Patients with other severe diseases, for example, cancers, renal disease, cardiovascular diseases, and autoimmune diseases were excluded from this study. This study has been approved by the Ethics Committee of The Central Hospital of Wuhan, and patients and healthy volunteers all signed the informed consent form. Plasma of the candidates was collected and stored at −80°C until needed.

### 2.1 Diagnosis of ACI

The diagnosis of ACI was under the guidance of 2014 American Heart Association/American Stroke Association (AHA/ASA) guidelines as previously described.^16^ Briefly, (a) Patient has the first ischemic stroke within 6 hours after the symptom onset; (b) results of CT or MRI analysis indicated that the patients have sudden occurrence of focal neurological deficits in ischemia lesions; (c) doctor has the adequate access to the clinical information of the patients. The subtype of ACI was divided based on the Trial of Org 10172 in Acute Stroke Treatment (TOAST),^17^ and patients were divided into four different subtypes: large artery atherosclerosis, small artery stroke, cardioembolism, and undetermined etiology.

### 2.2 Glasgow outcome scale

Glasgow outcome scale (GOS) has been applied for the evaluation of the short‐term prognosis of patients with ACI at day 30 as previously described. GOS score of 1: the patient was dead; GOS score of 2: the patient is at persistent vegetative state; GOS score of 3: the patient has severe disability; GOS score of 4: the patient has moderate disability; GOS score of 5: the patient only has low disability.

### 2.3 Real‐time quantitative PCR

Total RNAs were isolated from the plasma of the patients and healthy volunteers using TRIzol reagent (Invitrogen) according to the manufacture's protocols, and plasma levels of miR‐99a, miR‐99b, and miR‐100 were determined by Hairpin‐it™ miRNAs qPCR Quantitation Kit (purchased from GenePharma). PCR was conducted on ABI 7500 Real‐Time PCR System (Applied Biosystems) and U6 has been used as the internal control. The thermocycling profiles of the PCR were 95°C 30 seconds; 40 cycles of 95°C 5 seconds; and 60°C 30 seconds. The primers were synthesized by Sangon Biotech. The relative expression of miR‐99b was normalized to the level of U6 by 2^−ΔΔCt^ method.

### 2.4 Enzyme‐linked immunosorbent assay

To determine the plasma level of VEGF, commercially available an enzyme‐linked immunosorbent assay (ELISA) kit (a Biocalvin) was used. The ELISA assay was conducted according to the manufacturer's instructions.

### 2.5 Statistical analysis

The statistical analysis was performed by GraphPad version 6 (GraphPad Software). Data were presented as mean ± standard deviation (SD). Student's *t* test was applied for the comparison between two groups, and receiver operating characteristic curve (ROC) was drawn for the evaluation of the sensitivity and specificity of plasma level of miR‐99 for the diagnosis of ACI. Pearson analysis was conducted for the correlation analysis. *P* < .05 was considered as significant.

## 3 RESULTS

### 3.1 Clinical characteristics of the patients

Based on the TOAST criteria, the patients with ACI included 51 cases of large artery atherosclerosis (45.5%), nine cases of small artery stroke (8.0%), seven cases of cardioembolism (6.3%), and 45 cases of undetermined etiology (40.2%). The clinical information of the patients and the healthy controls were shown in Table 1. The age and gender showed no significant difference of the two groups, but the BMI, percentage of hypertension people, hyperlipidemia people, and diabetes people was significantly increased in ACI group compared with the control group.

**Table 1:** Clinical information of the patients and controls

	Control (n = 112)	ACI (n = 112)	*P*‐value
Age (Y)	63.42 ± 5.71	64.56 ± 6.03	.1477
Gender (Male/Female)	70/42	68/44	.7835
BMI	19.64 ± 2.39	23.11 ± 2.86	<.001
Hypertension (%)	35/112 (31.25%)	79/112 (70.54%)	<.001
Hyperlipidemia (%)	32/112 (28.57%)	64/112 (57.14%)	<.001
Diabetes (%)	15/112 (13.39%)	31/112 (27.68%)	<.001

### 3.2 Down‐regulation of miR‐99b in plasma of patients with ACI

**Figure 1:**
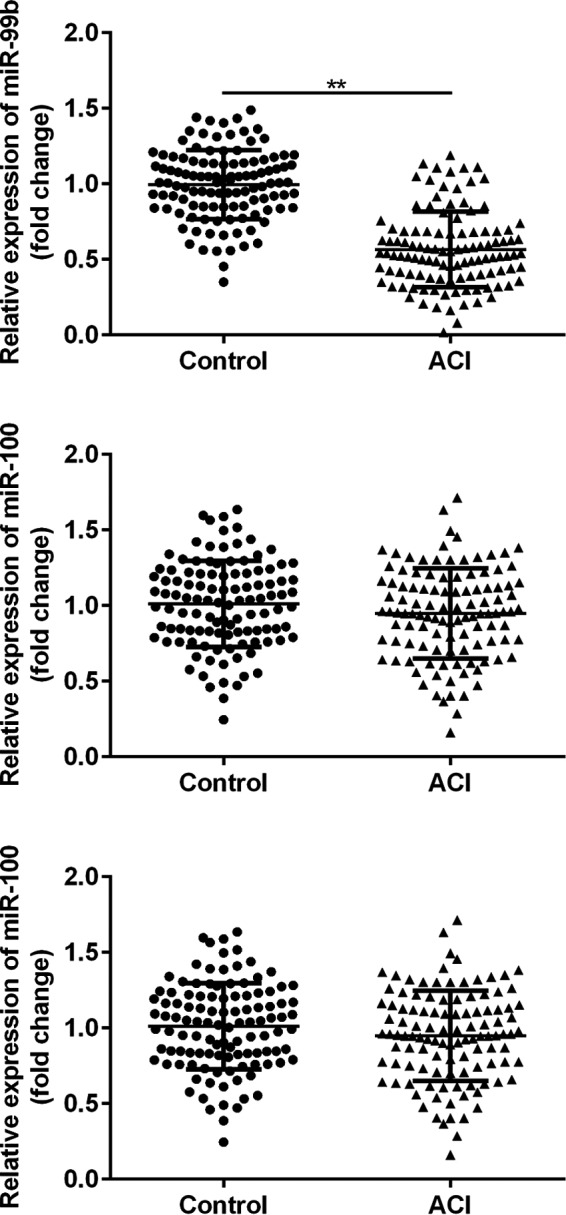
Comparison of the plasma levels of miR‐99a, miR‐99b, and miR‐100 in patients with ACI and the healthy volunteers. **P < .01

To begin with, the plasma levels of the miR‐99 family miRNAs, miR‐99a, miR‐99b, and miR‐100 were compared by RT‐qPCR methods. As shown in Figure 1, compared with the controls, the plasma level of miR‐99b was significantly decreased in patients with ACI (*P* < .01). On the other hand, the plasma levels of miR‐99a and miR‐100 showed no significant difference between the ACI and control group (*P* > .05).

### 3.3 miR‐99b may function as an early diagnostic marker for ACI

**Figure 2:**
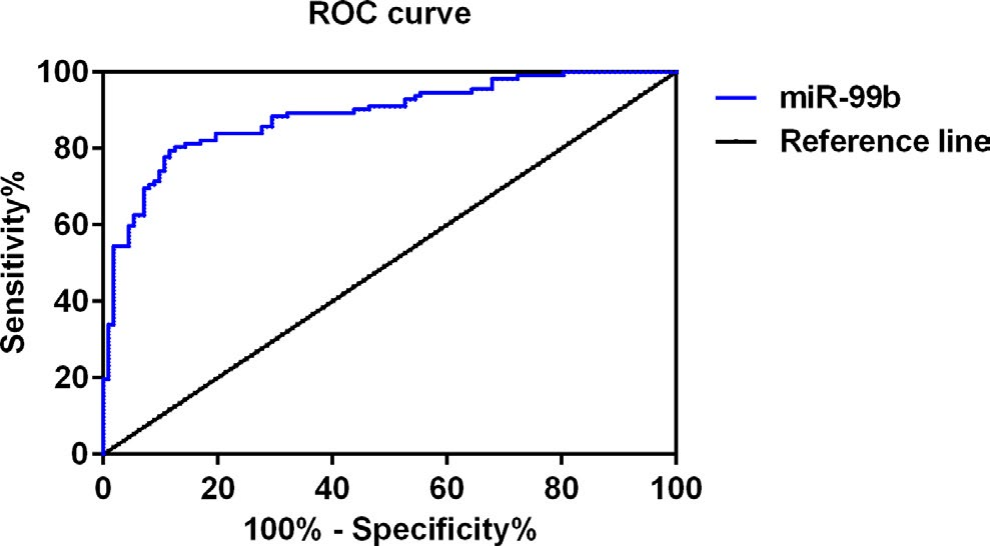
Results of receiver operating characteristic curve (ROC) to evaluate the sensitivity and specificity of miR‐99b for the diagnosis of ACI

Next, receiver operating characteristic curve (ROC) has been drawn to evaluate the sensitivity and specificity of plasma level of miR‐99b for the diagnosis of ACI. As shown in Figure 2, the area under curve (AUC) of miR‐99b was 0.8882 (95% confidence interval (CI), 0.8451‐0.9313), suggesting that plasma level of miR‐99b is a sensitive biomarker for the diagnosis of ACI.

### 3.4 miR‐99b may serve as a short‐term prognostic marker for ACI

**Figure 3:**
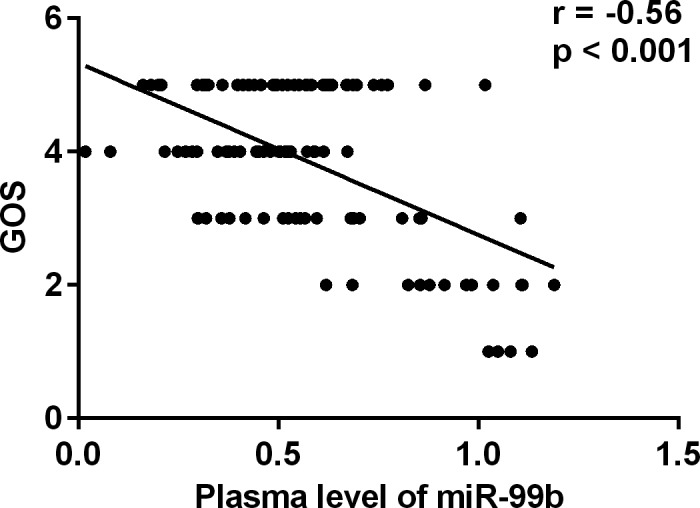
Correlation between the GOS and plasma levels of miR‐99b in patients with ACI

Moreover, the correlation between the plasma level of miR‐99b and the short‐term prognosis of the patients with ACI were analyzed. It was observed that the plasma level of miR‐99b was negatively correlated with the GOS of the patients (*r* = −.56, *P* < .001), suggesting that that plasma level of miR‐99b may function as a prognostic marker for the short‐term prognosis of ACI were analzed (Figure 3).

### 3.5 The level of miR‐99b was negatively correlated with the level of VEGF in plasma patients with ACI

**Figure 4:**
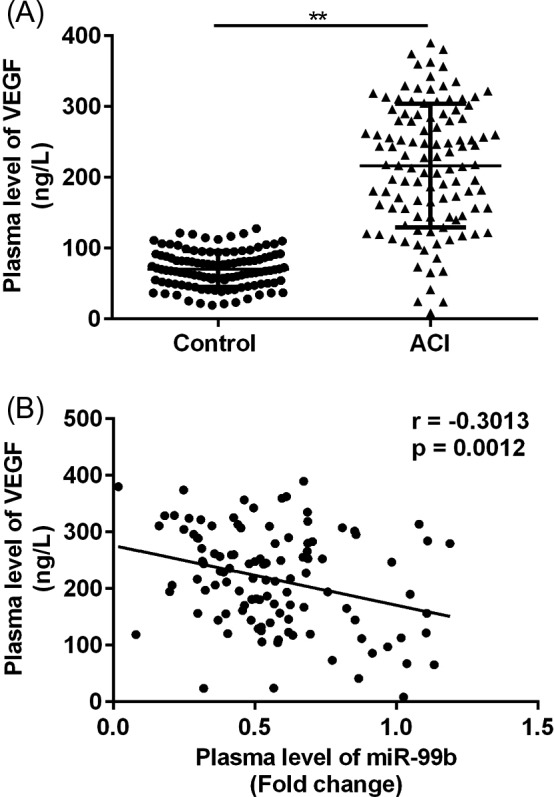
Correlation between the plasma levels of miR‐99b and VEGF in patients with ACI. A, Comparison of the plasma levels of VEGF in patients with ACI and the healthy volunteers. B, Results of correlation analysis. **P < .01

Finally, the correlation between the plasma level of miR‐99b and the angiogenic factor VEGF was analyzed. As shown in Figure 4, VEGF was significantly up‐regulated in the plasma of patients with ACI (*P* < .01), and the plasma levels of miR‐99b were negatively correlated with the levels of VEGF in patients with ACI (*r* = −.3013, *P* = .0012).

## 4 DISCUSSION

In the present study, we observed that miR‐99b was down‐regulated in plasma of patients with ACI compared with the healthy controls, and miR‐99b may serve as a potential diagnostic and prognostic marker for the early diagnosis and prediction of the clinical outcomes of patients with ACI.

Increasing evidence indicated that circulating microRNAs may serve as diagnostic markers in ACI. For example, Zhou et al reported that plasma levels of both miR‐21 and miR‐24 may function as early diagnostic markers at early stage of ACI^18^; Wang et al reported that miR‐210 may regulate the proliferation and apoptosis of the endothelial cells in ACI^19^; Zhang et al suggested that miR‐29b and miR‐424 may serve as prognostic markers and therapeutic targets for the treatment of ACI.^11^ The roles of miR‐99 family in cerebrovascular diseases have been discussed in some previous studies^13^, ^14^; however, it remains to be examined whether the plasma levels of the miR‐99 family miRNAs were changed in patients with ACI. In the present study, we observed that the expression level of miR‐99b was significantly down‐regulated in plasma of patients with ACI compared with the controls, and result of ROC analysis suggested that plasma level of miR‐99b is a sensitive biomarker for the diagnosis of ACI (AUC 0.8882, 95% confidence interval (CI), 0.8451‐0.9313). Interestingly, the plasma levels of the other two members of the miR‐99 family, miR‐99a and miR‐100 showed no significant difference between the control and ACI groups. Taken together, these results suggested that plasma levels of miR‐99b are a potential novel diagnostic biomarker for ACI.

Based on the result of previous studies, ACI can lead to serious sequelae; therefore, it is important to identify novel and sensitive prognostic markers.^11^ In the present study, the GOS of the patients was recorded for the evaluation of clinical outcomes, and we found that the plasma levels of miR‐99b were negatively correlated with the GOS of the patients, suggesting that miR‐99b may serve as potential prognostic markers in ACI.

Post‐infarction angiogenesis is an important event for the recovery of the brain function after cerebral infarction, and the roles of miRNAs in the process of angiogenesis after cerebral infarction have been reported previously. It has been observed that in cerebral infarction mouse models, miR‐145 can regulate the proliferation and migration of endothelial progenitor cells^20^; moreover, miR‐210 was able to activate the NOTCH signaling and improve the angiogenesis after acute cerebral ischemia.^7^ VEGF is known as an angiogenic factor, and in the present study, we observed that the plasma levels of VEGF were significantly up‐regulated in patients with ACI, which was consistent with previous findings.^21^, ^22^, ^23^ More important, we first reported that the plasma level of miR‐99b was negatively correlated with the level of VEGF in patients with ACI, suggesting that down‐regulation of miR‐99b may be involved in the process angiogenesis in brain tissue after cerebral infarction. Although VEGF has not been predicted as a direct of miR‐99b, we proposed that down‐regulation of miR‐99b after ACI may lead to the activation the angiogenic signaling pathway and increased expressions of some angiogenic signaling molecule (which are the direct targets of miR‐99b) and consequentially increased the expression of the downstream molecules, for example VEGF. However, the hypothesis still needs to be further investigated by in vitro cell studies and in vivo animal studies.

Our study has limitations. Patients with different subtype of ischemic stroke may have different short‐term prognosis; for example, it has been reported that patients with lacunar infarcts (a subtype of ischemic stroke) may have a better short‐term prognosis than other subtypes.^24^ Therefore, in future study, the prognostic roles of miR‐99 in the short‐term prognosis different subtypes of ischemic stroke should be analyzed. Also, hypertension is known as one of the main cardiovascular risk factors in lacunar stroke and atherothrombotic infarction.^25^ Therefore, in future study, the roles of miR‐99b in ACI should also be confirmed by comparing the expressions of miR‐99b in ischemic stroke patients with or without hypertension.

In conclusion, we observed that miR‐99b was down‐regulated in the plasma of patients with ACI and miR‐99b may serve as a diagnostic and prognostic marker in ACI, and miR‐99b may also participate in the process of angiogenesis after cerebral infarction. Our data have proposed the potential diagnostic and prognostic value of the plasma level of miR‐99b for the early diagnosis and treatment of ACI.

## CONFLICT OF INTEREST

The authors report no competing financial interests.

## References

[1] Liu Y , Li Y , Zhan M , et al. Astrocytic cytochrome P450 4A/20‐hydroxyeicosatetraenoic acid contributes to angiogenesis in the experimental ischemic stroke. Brain Res. 2019;1708:160–170. 30571981 10.1016/j.brainres.2018.12.023

[2] Wilkins SS , Akhtar N , Salam A , et al. Acute post stroke depression at a Primary Stroke Center in the Middle East. PLoS ONE. 2018;13(12):e0208708. 30571716 10.1371/journal.pone.0208708 PMC6301612

[3] Sasaki T , Yasuda T , Abe D , et al. A case of multiple cerebral infarction preceding acute exacerbation of idiopathic thrombocytopenic purpura. J Stroke Cerebrovasc Dis. 2019;28(3):789–791. 30553647 10.1016/j.jstrokecerebrovasdis.2018.11.026

[4] Luo L , Zhu M , Zhou J . Association between CTSS gene polymorphism and the risk of acute atherosclerotic cerebral infarction in Chinese population: a case‐control study. Biosci Rep. 2018;38(6):BSR20180586. 30341237 10.1042/BSR20180586 PMC6301210

[5] Cheng CY , Kao ST , Lee YC . Ferulic acid ameliorates cerebral infarction by activating Akt/mTOR/4E‑BP1/Bcl‑2 anti‑apoptotic signaling in the penumbral cortex following permanent cerebral ischemia in rats. Mol Med Rep. 2018;19(2):792‐804. 30569126 10.3892/mmr.2018.9737

[6] Huan Y , Chaoyang Z , Kai D , Chunhua S , Xin Z , Yue Z . Predictive value of head‐neck CTA combined with ABCD2 scale score for patients with cerebral infarction of vertebrobasilar transient ischemic attack (TIA). Med Sci Monit. 2018;24:9001‐9006. 30540723 10.12659/MSM.909470 PMC6299779

[7] Lou YL , Guo F , Liu F , et al. miR‐210 activates notch signaling pathway in angiogenesis induced by cerebral ischemia. Mol Cell Biochem. 2012;370(1–2):45‐51. 22833359 10.1007/s11010-012-1396-6

[8] Xiang W , Tian C , Lin J , et al. Plasma let‐7i and miR‐15a expression are associated with the effect of recombinant tissue plasminogen activator treatment in acute ischemic stroke patients. Thromb Res. 2017;158:121‐125. 28892656 10.1016/j.thromres.2017.09.004

[9] Ngeow J , Mester J , Rybicki LA , Ni Y , Milas M , Eng C . Incidence and clinical characteristics of thyroid cancer in prospective series of individuals with Cowden and Cowden‐like syndrome characterized by germline PTEN, SDH, or KLLN alterations. J Clin Endocrinol Metab. 2011;96(12):E2063‐E2071. 21956414 10.1210/jc.2011-1616 PMC3232626

[10] Wu H , Liu HY , Liu WJ , Shi YL , Bao D . miR‐377‐5p inhibits lung cancer cell proliferation, invasion, and cell cycle progression by targeting AKT1 signaling. J Cell Biochem. 2019;120(5):8120–8128. 10.1002/jcb.28091 30485528

[11] Zhang YZ , Wang J , Xu F . Circulating miR‐29b and miR‐424 as prognostic markers in patients with acute cerebral infarction. Clin Lab. 2017;63(10):1667‐1674. 29035456 10.7754/Clin.Lab.2017.170420

[12] Weng H , Shen C , Hirokawa G , et al. Plasma miR‐124 as a biomarker for cerebral infarction. Biomed Res. 2011;32(2):135‐141. 21551949 10.2220/biomedres.32.135

[13] Zhao H , Li G , Ma Q , et al. MicroRNA‐99a‐5p in circulating immune cells as a potential biomarker for the early diagnosis of ischemic stroke. Brain Circ. 2017;3(1):21‐28. 30276300 10.4103/bc.bc_1_17 PMC6126231

[14] He W , Chen S , Chen X , Li S , Chen W . Bioinformatic analysis of potential microRNAs in ischemic stroke. J Stroke Cerebrovasc Dis. 2016;25(7):1753‐1759. 27151415 10.1016/j.jstrokecerebrovasdis.2016.03.023

[15] Altintas O , Ozgen Altintas M , Kumas M , Asil T . Neuroprotective effect of ischemic preconditioning via modulating the expression of cerebral miRNAs against transient cerebral ischemia in diabetic rats. Neurol Res. 2016;38(11):1003‐1011. 27635859 10.1080/01616412.2016.1232013

[16] Kernan WN , Ovbiagele B , Black HR , et al. Guidelines for the prevention of stroke in patients with stroke and transient ischemic attack: a guideline for healthcare professionals from the American Heart Association/American Stroke Association. Stroke. 2014;45(7):2160‐2236. 24788967 10.1161/STR.0000000000000024

[17] Adams HP Jr , Bendixen BH , Kappelle LJ , et al. Classification of subtype of acute ischemic stroke. Definitions for use in a multicenter clinical trial. TOAST. Trial of Org 10172 in Acute Stroke Treatment. Stroke. 1993;24(1):35‐41. 7678184 10.1161/01.str.24.1.35

[18] Zhou J , Zhang J . Identification of miRNA‐21 and miRNA‐24 in plasma as potential early stage markers of acute cerebral infarction. Mol Med Rep. 2014;10(2):971‐976. 24841240 10.3892/mmr.2014.2245

[19] Wang J , Zhang Y , Xu F . Function and mechanism of microRNA‐210 in acute cerebral infarction. Exp Ther Med. 2018;15(2):1263‐1268. 29434712 10.3892/etm.2017.5577 PMC5774459

[20] Chen R , Chen S , Liao J , Chen X , Xu X . MiR‐145 facilitates proliferation and migration of endothelial progenitor cells and recanalization of arterial thrombosis in cerebral infarction mice via JNK signal pathway. Int J Clin Exp Pathol. 2015;8(10):13770‐13776. 26722607 PMC4680552

[21] Liu D , Tang ZY , Hu ZJ , Li WW , Yuan WN . MiR‐940 regulates angiogenesis after cerebral infarction through VEGF. Eur Rev Med Pharmacol Sci. 2018;22(22):7899‐7907. 30536336 10.26355/eurrev_201811_16416

[22] Kim E , Yang J , Park KW , Cho S . Inhibition of VEGF signaling reduces diabetes‐exacerbated brain swelling, but not infarct size, in large cerebral infarction in mice. Transl Stroke Res. 2018;9(5):540‐548. 29290003 10.1007/s12975-017-0601-z PMC6057840

[23] Zhang B , Wang D , Ji TF , Shi L , Yu JL . Overexpression of lncRNA ANRIL up‐regulates VEGF expression and promotes angiogenesis of diabetes mellitus combined with cerebral infarction by activating NF‐kappaB signaling pathway in a rat model. Oncotarget. 2017;8(10):17347‐17359. 28060742 10.18632/oncotarget.14468 PMC5370045

[24] Arboix A , Marti‐Vilalta JL . New concepts in lacunar stroke etiology: the constellation of small‐vessel arterial disease. Cerebrovasc Dis. 2004;17(Suppl 1):58‐62. 14694281 10.1159/000074796

[25] Arboix A , Roig H , Rossich R , Martinez EM , Garcia‐Eroles L . Differences between hypertensive and non‐hypertensive ischemic stroke. Eur J Neurol. 2004;11(10):687‐692. 15469453 10.1111/j.1468-1331.2004.00910.x

